# Experimental Verification of the Elastic Response in a Numeric Model of a Composite Propeller Blade with Bend Twist Deformation

**DOI:** 10.3390/polym13213766

**Published:** 2021-10-30

**Authors:** Sondre Østli Rokvam, Nils Petter Vedvik, Lukas Mark, Eivind Rømcke, Jon Schawlann Ølnes, Luca Savio, Andreas Echermeyer

**Affiliations:** 1 Department of Mechanical and Industrial Engineering (MTP), Norwegian University of Science and Technology (NTNU), 7491 Trondheim, Norway; nils.p.vedvik@ntnu.no (N.P.V.); lmark@ualberta.ca (L.M.); Eivindrom@gmail.com (E.R.); jon.olnes@gmail.com (J.S.Ø.); andreas.echtermeyer@ntnu.no (A.E.); 2 SINTEF Ocean, Otto Nielsens vei 10, 7052 Trondheim, Norway; luca.savio@sintef.no; 3 Kongsberg University Technology Centre “Performance in a Seaway” at NTNU, N-7491 Trondheim, Norway

**Keywords:** composites, propellers, bend-twist, FEA, DIC, experimental verification

## Abstract

Adaptive composite propeller blades showing bend twist behaviour have received increasing interest from hydrodynamic and structural engineers. When exposed to periodic loading conditions, such propellers can be designed to have higher energy efficiency and emit less noise and vibration than conventional propellers. This work describes a method to produce an adaptive composite propeller blade and how a point load experiment can verify the predicted elastic response in the blade. A 600 mm-long hollow full-size blade was built and statically tested in the laboratory. Finite element modelling predicted a pitch angle change under operational load variable loads of 0.55°, a geometric change that notably compensates for the load cases. In the laboratory experiment, the blade was loaded at two points with increasing magnitude. The elastic response was measured with digital image correlation and strain gauges. Model predictions and experimental measurements showed the same deformation patterns, and the twist angle agreed within 0.01 degrees, demonstrating that such propellers can be successfully built and modelled by finite element analysis.

## 1. Introduction

Large ships often use propellers for propulsion and manoeuvring. In some manoeuvring operations, the propeller blades may operate in loading conditions where they are unequally loaded [1]. When the propeller rotates in such operating conditions, the propeller blades move through these different loads, which causes the load on each propeller blade to vary throughout each revolution. Such periodic load variation on the blades can cause undesired inefficiencies, cavitation, vibrations and noise (harmful towards marine life) [2,3].

While ship propeller blades are traditionally made of rigid metals, propeller blades may be designed using composite materials such as fibre-reinforced plastics (FRP). The anisotropic nature of FRP allows for designing a bending induced twist deformation into the propeller blade through the FRP surface ply stack layup [4,5,6]. The bend-twist deformation can function as an immediate pitch adjustment which mitigates the periodic load variations [1,2,6,7,8,9,10,11]. Other researchers have designed and manufactured FRP propeller blades with passive adaptive bend twist deformation [4,5,9,10,11,12]. For more information about periodic load variation on propeller blades and the mechanics of how a pitch adjustment physically mitigate load variations, refer to [1,7,9,10,11].

In recent years, there has been an increased focus on the optimisation of composite propellers from both structural and marine engineers points of view. Several different design concepts for twisting propeller blades have been looked into [1,4,5,6,9,10,11,12,13,14]. In addition, hydrodynamic performance and modelling methods to establish hydrodynamic performance are being mapped [14,15,16,17,18,19,20,21]. Most of this work has been undertaken without experimental verification into whether the explored designs are producible with the intended deformation characteristics and elastic response. However, real-life prototypes have been manufactured for experimental verification in some cases. The studies, [4] (full-scale) and [22] (model scale), performed point load experiments to explore the deformation characteristics of their produced prototypes. Reference [23] examined model-scale blades considering the fluid loading and structural elastic response. Measurement techniques for detecting the deformation characteristics were developed [20]. In addition, full-scale composite propeller prototypes were tested on vessels [17]. Studies considering strength and durability were undertaken for the prototypes [24]. Authors even talk about an ongoing technological breakthrough as the sum of these works accumulates [25].

The point load static test has been considered helpful as it would probably be applied in a manufacturing line for commercial composite propellers where quality and property checks must be performed at satisfactory intervals in the production. While both these studies [4,22] showed a good match between measured and predicted deflection of the blade, only [22] compared blade bending and blade twisting.

**Figure 1:**
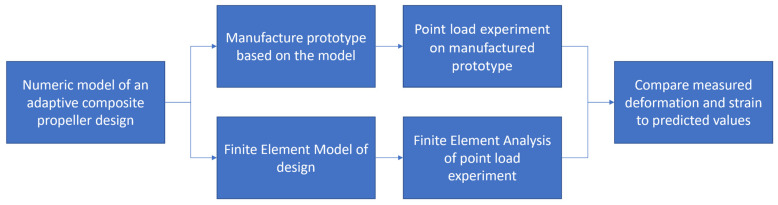
Block diagram to visualise the structure of the research in this paper.

This paper investigates whether the predicted blade deflection and twist match observed results. First, the propeller blade design, experiment design and FEA of the experiment are described. Then the manufacturing process and execution of the investigation is described. Next, deformation measurements and especially the change of pitch angles are described and compared to the FEA. The research structure in the paper is shown in Figure 1. Finally, a discussion of the procedure and the results follows before conclusions are drawn.

## 2. Materials and Methods

This paper investigates a propeller blade model made of carbon fibre reinforced epoxy, hereafter referred to as CFRP (carbon fibre-reinforced polymer). The material layup of the propeller blade is based on designs proposed in earlier works [1,9,10]. A simple point load experiment, both modelled in a finite element analysis (FEA) and staged physically, was proposed to evaluate whether a prototype based on the design exhibits the predicted bend-twist deformation. Based on Kumar and Wurm [4] and Maljaars, Kaminski, and den Besten [22], it is expected to find comparable deformation between experimental results and numeric modelling. If the deformations predicted by the numerical model and the experimental measurements of the point load configuration correlate, it is reasonable to expect that the numeric model of the propeller blade can also accurately predict the deformations when fluid loads are applied.

The propeller blade prototype was manufactured using a CFRP split mould. A 3D scan of the prototype was compared with the propeller blade 3D model for geometric verification. The manufactured propeller blade prototype was mounted to a test rig made out of a thick steel plate to carry out the experiment. Two point loads were applied to the blade by hanging metal weights from loading points chosen to make the point load case imitate the load in the periodic load case. The resultant blade deformation was measured with digital image correlation (DIC) technology, which tracked the deformations on one side of the blade. In addition, some strain gauges were used in a few places on the blade to evaluate the material response in the prototype.

**Figure 2:**
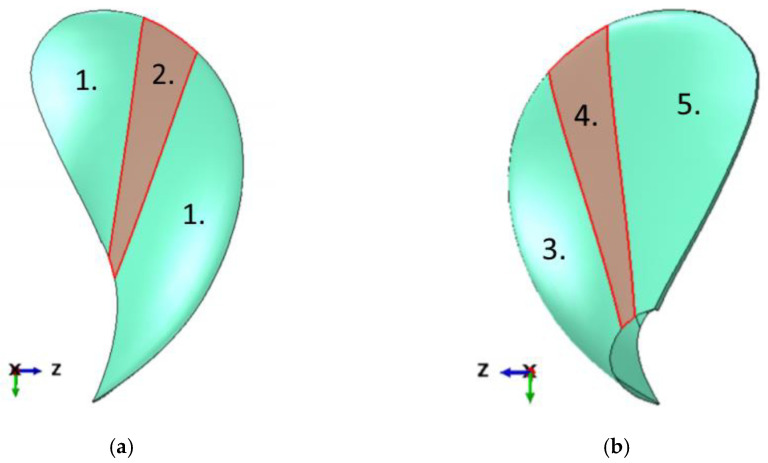
The investigated fibre-reinforced polymer (FRP) material layup design. The stacking arrangement of the plies in the layup is given in Table 1. (**a**) Suction side of the design; (**b**) pressure side of the design.

The CFRP product series XPREG (carbon fibre prepreg material, Easy Composites Ltd, Stoke on Trent, England) was used to build the prototype. The propeller blade design in the numeric model is shown in Figure 1, and the stacking details of the layup is given in Table 1. The FRP layup had woven plies used as backing plies, surface plies, and in the stiffening patches indicated by the red area in Figure 2. In addition, unidirectional (UD) plies at specific angle orientations were used in the design.

The surface plies are 0.22 mm, the backing plies are 0.46 mm, and the unidirectional plies are 0.3 mm, according to the XPREG product series datasheets [26,27]. The local stiffening patch in red and the unidirectional plies contribute towards bend-twist deformation [1,9,10,11]. While some material properties are given in the same datasheet, it was chosen to measure these experimentally. The results are shown in Table 2 [9,10,11].

The CFRP layup of the investigated propeller blade design was modified slightly by adding tapering between plies, based on conventional composite engineering practice. In addition, practical modifications were undertaken to obtain the experimental version of the prototype; most importantly, adding a root structure on the blade. In the original propeller blade design, the bottom edge of the blade was fixed in place. To make a prototype design that allows for a similar fastening of the blade, a root structure that allows the blade to be mounted to a stiff test rig was added. To simulate that the bottom of the blade in Figure 1 is fixed in place, the root structure must be much stiffer than the blade itself. Therefore, the root was made in a quasi-isotropic layup consisting of 14 backing plies, two surface plies, and four UD plies.

**Figure 3:**
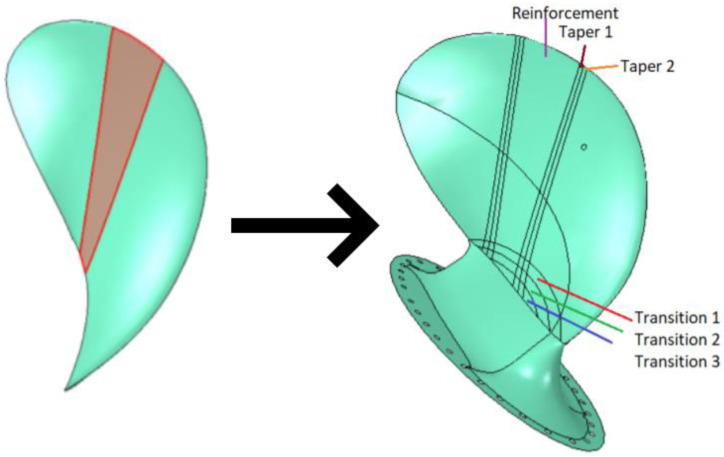
Modifications of the original design so the propeller blade structure can be tested in a point load experiment.

The surface plies and UD plies extend into the blade to avoid a weak connection between the blade and root. The extra plies in the root cause the laminate in the root structure to be 2–3 times thicker than the blade surface laminates (depending on which side of the blade the root is compared to), making for a sharp transition in stiffness between the root and the blade. In addition, tapering transitions were added in good composite engineering practice to limit stiffness discontinuities and weak points in these transitions. All modifications from the original FE model to the experimental version of the blade are shown in Figure 3.

After these modifications, the prototype was a bit more than 600 mm in height. The top 400 mm is the blade structure, and the bottom 200 mm consists of the root structure.

### 2.1. Design of the Experiment

Ideally, the prototype blade would be loaded with the operational periodic load case, and the resulting deformations would be measured. However, loading the propeller blade with an operational fluid load case was considered too complex to recreate in an experiment with a prototype this size. Therefore, a representative point load experiment was chosen. (In the project this paper is a part of, FleksProp, a fluid load is applied to a model scale bend-twist propeller prototype in a cavitation tank. The findings from this investigation will be published in separate works).

The resultant force that counteracts the pressure distribution simulating the periodic load case was examined to find a point load scenario representative of the original periodic load variation. The resultant force on the blade was split into the force components in the X, Y and Z directions. The X-direction was the travel direction, Y the radial direction of the blade, and the Y and Z plane the plane of rotation. The force component in the Y direction was much smaller than the other force components throughout the periodic load case. Therefore, the Y component was deemed negligible, leaving X and Z as the interesting plane.

**Figure 4:**
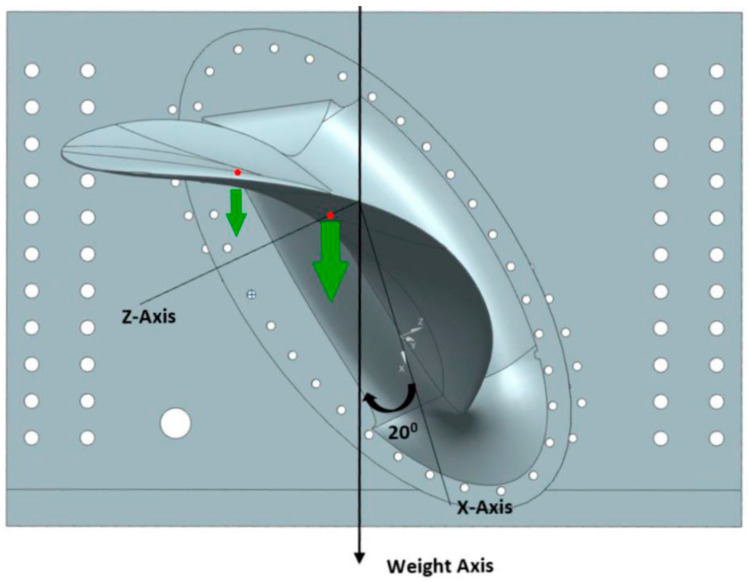
The setup of the experiment in a CAD model. The prototype is mounted to a steel test rig. The red dots indicate the loading points. Figure from [9].

The blade was suspended in the XZ plane by mounting the propeller blade prototype to the vertical steel test rig, as shown in Figure 4.

The test rig and the root were designed so that vertical loads on the blade would act in the XZ plane. Since the force components in the X and Z direction vary throughout the periodic load case, the angle shown in Figure 4 changes throughout the periodic load case. To find the angles corresponding to the interesting loads, the force components in the X and Z-direction were quantified for maximum and minimum loading in an interesting periodic load case from earlier work [1]. The force direction was found to vary between 34.3° for the least loaded blade position and 21.8° for the most loaded blade position, as shown in Table 3.

The maximum periodic load was deemed to be the most interesting load case and is focused on in this paper. To mimic the maximum loading in the periodic load case, the propeller prototype was mounted so that vertical loads would have a resultant angle of 20°, as shown in Figure 4.

The load point locations were set in collaboration with hydrodynamic researchers in the Fleksprop project that this paper is a part of. The main loading point was chosen to be at propeller radius 0.7 and chord line length 0.25, indicated by the right red dot in Figure 4. This point was close to the centre of pressure on a foil. In addition, a secondary load point at radius 0.9 and chord line length 0.25 was added to distribute the load more. The secondary load point is shown as the left red dot in Figure 4. The load was distributed with 25% of the load on the secondary load point and 75% on the main load point.

(When the experiment was undertaken, the propeller prototype was also tested in a configuration mimicking the minimum periodic load case, and other loading point possibilities were tested. Neither of these are included in this paper. Refer to [9,10] to examine the work covering these variants of the experiment.)

### 2.2. Finite Element Analysis (FEA) of the Experiment

**Figure 5:**
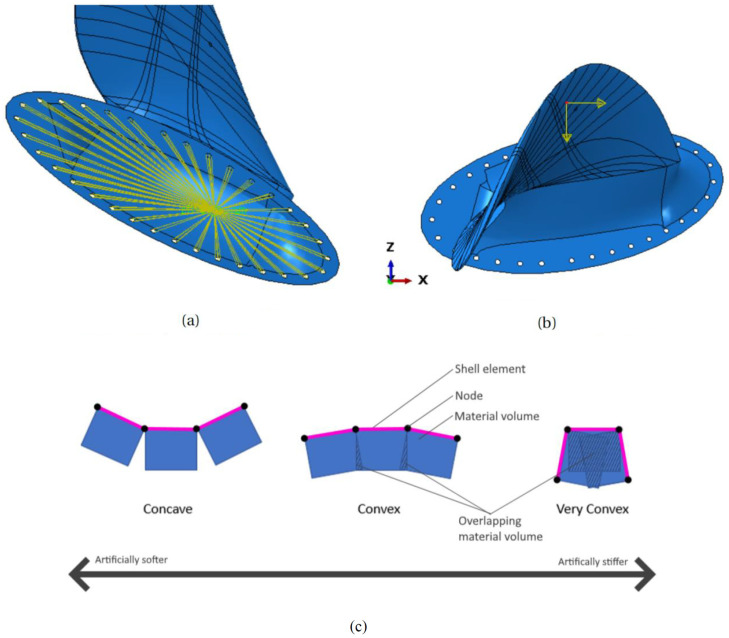
Visualisation of finite element analysis (FEA) aspects in the simulation of the experiment. (**a**) Boundary condition with all bolt holes fixed in place, Figure from [10]; (**b**) load vector component on r = 0.7. Figure from [10]; (**c**) material volume inconsistencies when using shell elements.

The experiment was numerically modelled with linear solutions in an FEA using the software Abaqus 2017 (finite element analysis software, 3DS Dassault Systèmes, Vélizy-Villacoublay, France). The boundary condition (BC) should lock all degrees of freedom in the model’s bolt holes to resemble a rigid connection to the test rig. Such a BC was achieved by connecting all displacements and rotations in the bolting holes nodes to an Abaqus reference with a Multi-Point Constraint (MPC) and then locking all degrees of freedom in the reference point, shown in Figure 5a. The point loads were applied to the nodes at the loading points as force vectors, as shown in Figure 5b. The load vectors were assigned to nodes on the side facing down in Figure 4.

The numeric model was based on shell elements, which are efficient for thin shell structures. However, joint and overlapping material volumes, which may add local artificial stiffness, is an issue when modelling shell elements with shell offset, as illustrated in Figure 5c. The effect is especially pronounced at sharp corners and convex surfaces like the thin trailing edge and the sharp corners on the back of the root. The same effect causes artificially lower stiffness in shell elements on concave surfaces, giving a lower local stiffness.

## 3. Manufacture and Experimental Setup

This section covers the steps and choices made in the process of bringing the planned experiment into reality. This includes the production of the prototype with a geometric verification, details regarding the staging of the experiment with the loading mounting of the prototype and the measurement techniques. For a more thorough description of these details, refer to [9,10].

### 3.1. Manufacture of Composite Prototype

A two-part split mould approach was chosen to produce the prototype from the 3D model with the available production equipment. A CFRP similar to the CFRP used for the prototype was used for the mould. Using similar materials in the mould and produced component facilitates stable geometry during the heated curing since the materials have a similar thermal expansion coefficient. The split line in the casted prototype was chosen to follow the propeller blade’s leading edge and trailing edge.

**Figure 6:**
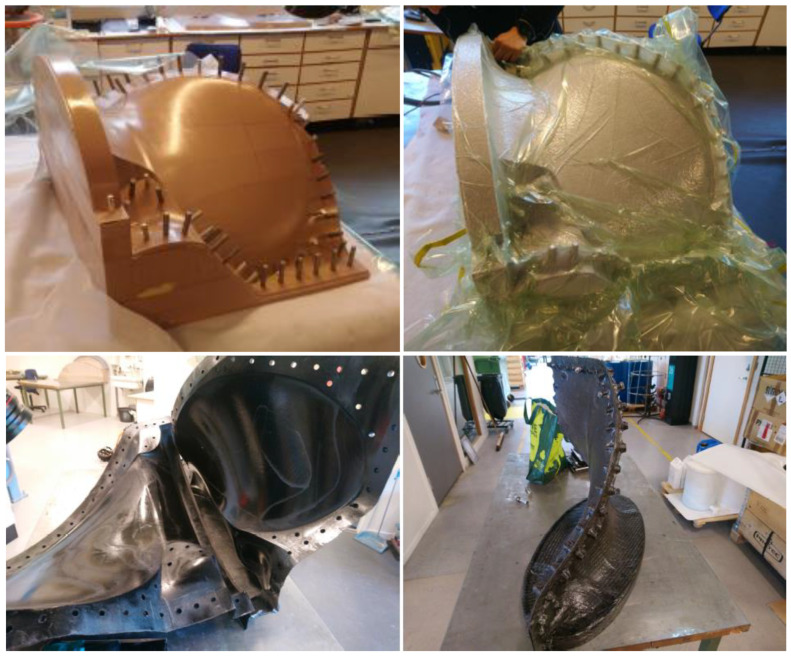
Top left: Mould pattern with alignment pins. Top right: Vacuum bagged during debulking of plies and curing of mould half. The finished mould is shown in the bottom two pictures. Photos from [10,11].

The two mould halves were cast on positive patterns milled out of a tooling board material called Renshape BM5460 (tooling board, Huntsman Corporation, Texas, United States). Before casting, metal pins that align the two mould halves during casting were added. The finished pattern, the mould construction and the finished mould is shown in Figure 6.

After the mould halves were produced, the CFRP layup was applied to each mould half. The plies were arranged with the aid of hand tools, and the fibre’ orientation in each ply was confirmed with visual inspections. This process was undertaken as meticulously as possible since accurate positioning of the fibre direction in the plies is of the utmost importance to obtain the intended anisotropic properties in FRP components [28]. Finally, the layup was debulked using vacuum and hand tools after each added ply to minimise voids and imperfections in the cast.

**Figure 7:**
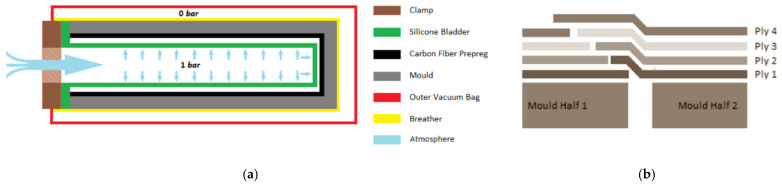
(**a**) Split mould arrangement during casting. (**b**) To the right is an illustration of tapered FRP in the joint of the mould seam shown. Illustrations from [10].

After the material layup was added on both mould halves, the mould halves were closed around a custom casted silicone bladder. The silicone bladder allowed for an airtight seal, giving even atmospheric pressure on the FRP layup from inside the mould, as shown to the left in Figure 7. On the leading-edge mould seam, where the two propeller blade halves meet, the two halves were connected with a tapered joint, illustrated to the right on Figure 7.

**Figure 8:**
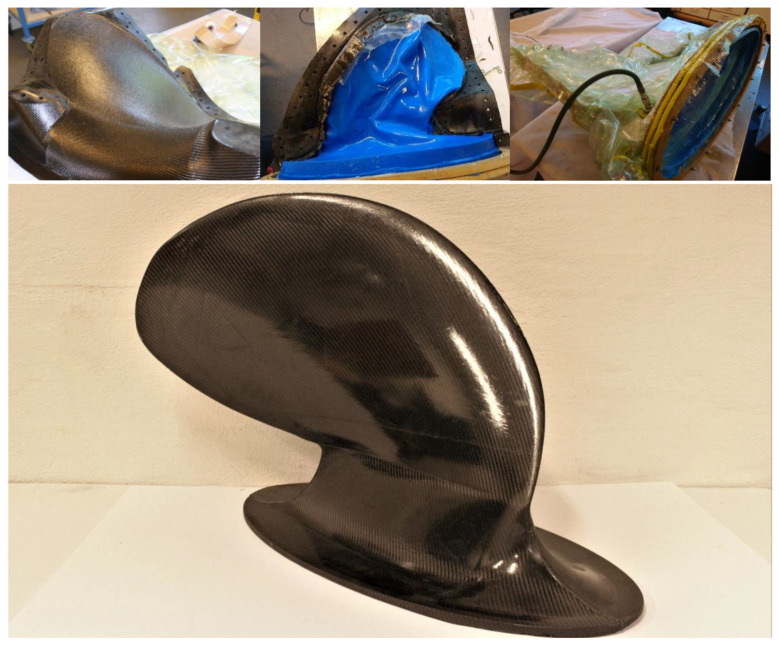
CFRP layup on a mould half, mould half with layup and silicone bladder, the assembled mould before oven curing cycle and the finished cast after demolding. Photos from [9].

The CFRP product series used should be cured under heat in a vacuumed or autoclaved state. As an autoclave was not available, an oven and vacuum pump were used in the curing process. The applied material layup on a mould half, the silicone bladder and the assembled vacuumed mould are shown in the top row of photos in Figure 8. After the FRP’s curing cycle in the oven, the cast was demoulded. The finished casted prototype is shown in the bottom photo in Figure 8.

The casted prototype was examined with Atos 5 (High-Precision 3D scanner, Coventry, England) to make a 3D model of the prototype. The scanned 3D model of the cast was compared to the original 3D model of the prototype to identify any geometric deviations in the produced prototype.

### 3.2. Load Case and Measurements

After the casted prototype was scanned, some final measures were taken before the experiment could commence. First, a series of holes were drilled in the root of the cast to mount the propeller blade to the rigid test rig. Then, the prototype was bolted to the test rig in a 20° orientation to mimic the most loaded blade position.

It was decided to apply the load case to the propeller blade by hanging weights. In the periodic load case, the total load on each propeller blade averages around 20 kN ≈ 2000 kg. With the available weights, and because the weights had to be added by hand, the total load in the experiment was limited to 500 kg. First, the location of the loading points was identified with the aid of hand tools. Then, holes were drilled in the blade at the loading point locations. Finally, a static climbing rope was fed through the holes in order to hang the weights. A barrel knot was used on the rope to make sure the rope did not accidentally feed through the hole during the experiment. On the 0.7 radius load point, a 3D printed fitted plastic washer was added between the prototype and the knot to diminish possible stress concentrations.

**Figure 9:**
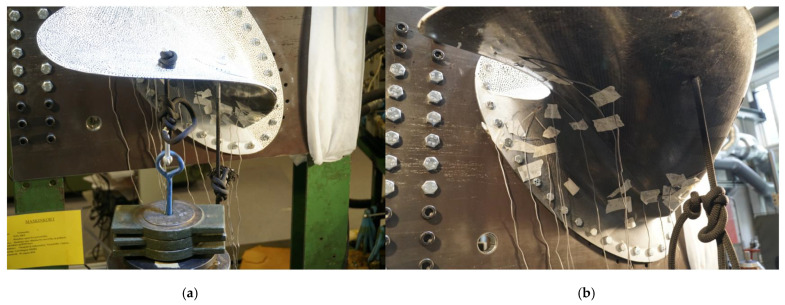
(**a**) The load was applied by hanging metal weights. The CFRP propeller blade is loaded with 500 kg, 375 kg at the 0.7 radius line and 125 kg at the 0.9 radius point in the left photo. (**b**) The strain gauges were attached on the blade and the root.

Figure 9a,b shows the prototype mounted to the rigid steel test rig with weights suspended from the load points and attached measuring devices. The strain gauges were connected to HBM QuantumX MX1615B (data acquisition system, HBM Inc., Marlborough, MA, USA), which sent the data to a computer with Catman^®^ AP (data acquisition software, HBM Inc., Marlborough, MA, USA) to log the strain data continuously throughout the experiment. The strain gauges used were the type FLA-6-11-3L (strain gauge, Sokki Kenkyujo Co., Ltd., Tokyo, Japan) with a gauge length of 6mm.

During the tests, the load was increased incrementally by adding weights to the load points in 100, 50, 20 and 10 kg increments.

**Figure 10:**
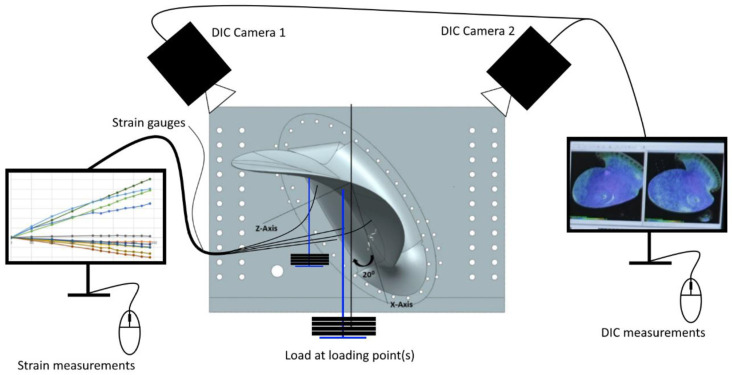
Schematic of experiment design. The propeller blade is incrementally loaded while strain and digital image correlation (DIC) measurements are logged.

DIC snapshots were taken after each load increment to measure the deformation from the load. The DIC measured the 3D deformation visible on the produced prototype’s pressure side (the side facing up in Figure 9 a). The preparations for performing these measurements included painting the pressure side white before a black dot speckle with 2–3 mm dots were added with permanent black markers. Then, two cameras were set up, as shown in Figure 10, to track the displacement of the speckle pattern on the propeller blade surface. Using the DIC cameras (Prosilica GT, CorrelatedSolutions, Irmo, United States), measurements were taken with the software Vic-Snap 8 (DIC camera software, CorrelatedSolutions, Irmo, United States). The DIC post-processing necessary to calculate the deformation visible in the snapshots, was undertaken with the software Vic-3D 8 (DIC analysis software, CorrelatedSolutions, Irmo, United States). A schematic of the experiment showing the test rig, loading, and measurement equipment is given in Figure 10.

## 4. Results and Numerical Predictions

In this section, the experimental measurements are compared to the corresponding predictions. First, the manufactured prototype is described and compared to the 3D model for geometric verification of the prototype. Then, the DIC measurements are presented in full and compared to the FEA, and a load-max displacement graph is shown before the deformation characteristics, the bending deflection and twist of the blade, in the FEA and experiment, are evaluated and compared.

Finally, the strain measurements and numeric predictions are given to evaluate the material response in the experiment to the FEA.

### 4.1. Manufactured Propeller Blade Prototype

A hollow CFRP propeller was cast. After curing, the propeller blade was easily demoulded from the mould, but the silicone bag tore upon removal, leaving a part of the bag stuck inside the prototype. The prototype weighed 7.5 kg. A visual inspection of the prototype shows a successfully produced propeller blade with two minor imperfections. First, some harmless minuscule voids in various places, and secondly, close to the tail of the root, the small part of the silicon-rubber bag poked through the split-seam on the prototype.

**Figure 11:**
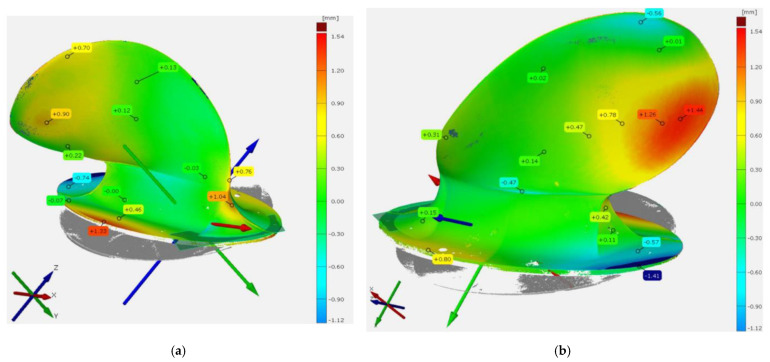
A 3D scan of the prototype is compared with the 3D model on which the prototype was based. The comparison was made with the software GOM Inspect Suite and shows that the geometry matches well in most areas, but some deviations are present. (**a**) Suction side view of prototype. (**b**) Pressure side view of prototype.

Figure 11 shows the comparison between the 3D scan of the prototype and the 3D model. It can be seen that the geometry mostly matches within an error margin of 0.3 mm. However, the geometries do deviate, and an extra thickness of 2.3 mm was found close to the tail. In this area, the 3D model had a thickness of 5–10 mm while the material layup’s thickness was 8.42 mm, plus the stuck silicone rubber bag. Therefore, it seems the maximum deviation could be due to too much material in a too small volume.

The deviations in the prototype were not expected to affect the prototype’s structural response and deformation characteristics notably, and the production is viewed as a success. However, a better surface finish and a higher tolerance production method will be needed to produce realistic commercial-grade blades to be used in operation.

### 4.2. Comparing Digital Image Correlation (DIC) Measured Deformation Characteristics and Numerical Predictions

After post-processing the DIC measurements and the FEA, the colour contour plots in Figure 11 were produced. The plots show the deformation in the prototype on a global scale in coordinate system axes. In the DIC plots to the right in Figure 11, it is clear that some parts of the blade are not plotted. Considering both the size and the complex geometry of the prototype, it is not surprising that some data were lost, probably due to effects like glare or the deformation causing the speckle pattern to move out of focus. The big white hole in the DIC data is caused by the washer distributing the applied load. Nonetheless, it is possible to compare the DIC measured deformation to the corresponding FEA. As seen on the magnitudes and the deformation contours, the model shows good agreement on the global scale.

**Figure 12:**
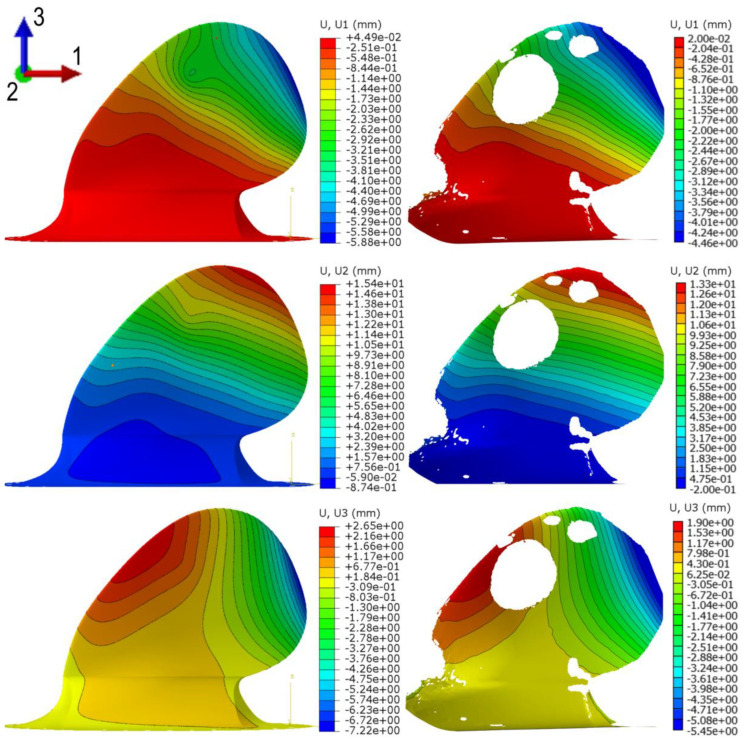
Global displacements in different directions observed on the DIC measurements (**right**) and the corresponding FEA deformation plot (**left**). The plot shows the displacements under maximum load, 375 kg applied at radius 0.7 and 125 kg applied at radius 0.9; a total load of 500 kg.

Figure 12 addresses the deformation characteristics measured qualitatively, but an exact comparison needs to be done to investigate quantifiable deformation characteristics further. The task was approached by comparing similar areas in the DIC and FEA.

**Figure 13:**
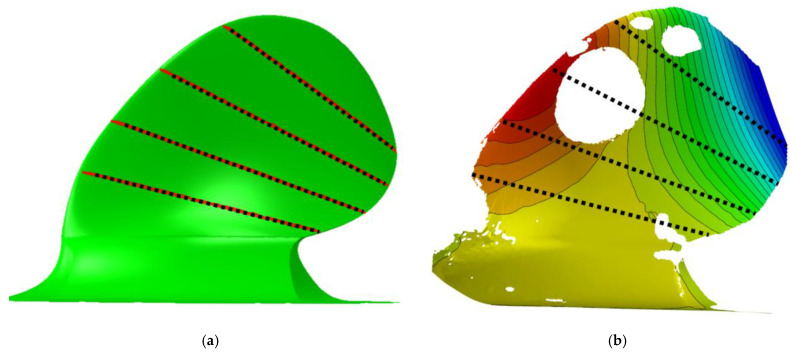
The displacements on the path lines from the blade’s trailing edge to the leading edge of the propeller blade were used to observe the quantifiable twist deformation characteristics, bend and twist unambiguously. (**a**) Analysis along a line in the FEA. (**b**) Analysis along a line in the DIC.

**Figure 14:**
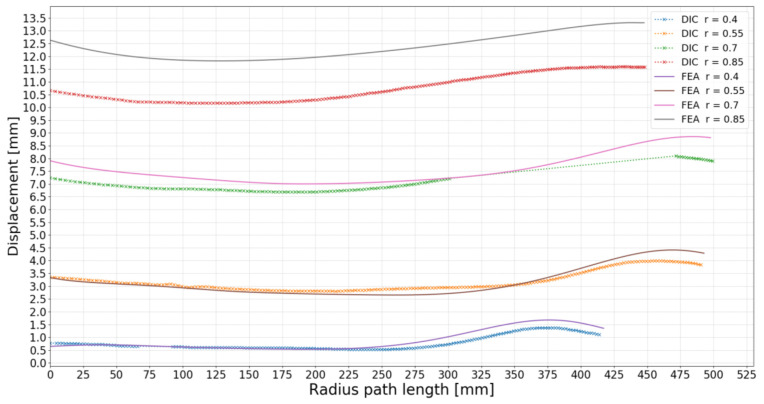
Line plot of the displacement at the lines in Figure 13 for FEA and experiment comparison. The graph starts at the tail point on the left to the nose point on the right. Some data were missing in the DIC measurements due to the DIC not capturing the deformation of the plastic washer used to distribute the load.

As seen in Figure 13, four lines were picked in similar locations in the DIC and the FEA to plot the displacements of these lines for comparison. The lines span from the blade’s tail to the nose at the normalised propeller radii 0.4, 0.55, 0.7 and 0.85. When plotted, the deflection at these lines can be observed unambiguously in Figure 14.

The displacement of each line was subdivided into average blade deflection and blade twist in degrees at the inspected radii. As mentioned earlier, the deformation characteristics of the root, the 0.4 radius line, are subtracted from the other to only focus on the blade.

The displacements in the prototype were divided into the deformation modes bending deflection and blade twist. If no twist is present, the displacement at the trailing edge and the leading edge will be the same. The twist and deflection characteristics are given for each investigated radii in Table 4.

The blade deflection was calculated as the average displacement for an inspected line, and the twist was calculated as the difference in displacement at the trailing and the blade’s leading edge. In Table 4, it is shown that the FEA show a twist of 0.318° and the DIC a twist of 0.307° (when subtracting the twist at radius 0.4). These values indicate that the blade is modelled quite accurately.

From Figure 14, it can be seen that the blade’s leading edge (on the right side) deflect more than the trailing edge in both the DIC and FEA. This means that the blade deforms to a positive pitch change, the opposite of the sought-after negative pitch change that would mitigate the periodic load variations. As the numeric prediction and experiment match, the positive twist reflects that the chosen load case was not mimicking the most loaded periodic load as expected. One possibility is that the centre of pressure tends to move back on the foil when the blade is exposed to the maximum loaded periodic load as the blade stalls, which was not considered when finding the load points.

### 4.3. Mechanical Response—Strains

The evaluation of how well the blade’s FEA shell model captured the prototype’s actual mechanical response was undertaken by comparing the predicted and measured strains. The strain values predicted in the FEA were found by examining elements close to where the strain gauges had been placed on the prototype. Strain gauge 5 was placed on the tail, slightly below the area with extra thickness, and strain gauge 6 was located on the root and blade transition. The two last strain gauges were placed on the root, close to the back of the blade. All the strain gauges were placed on the suction side, facing down in Figure 9 and Figure 10.

**Figure 15:**
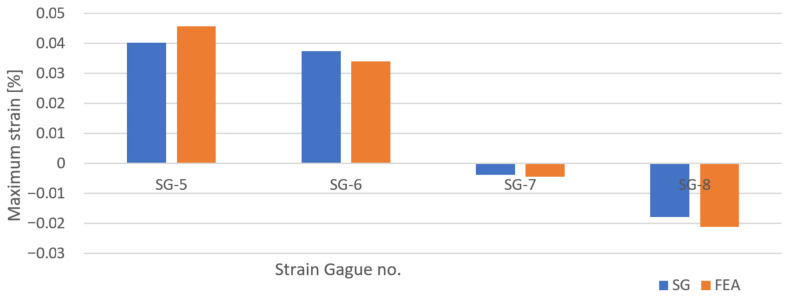
Comparison between strain gauge data and predictions in the FEA. It is seen that the FEA overestimates the strain in general but underestimates the strain at strain gauge 6, located at a ply overlapping joint.

The comparison of strains in the experimental results and FEA for the experiment is shown in Figure 15. The overall agreement between FEA simulations and experiments is good, within 100 microstrain. The FEA overestimates the strain for the strain gauges 5, 7 and 8. However, an opposite observation is found in the strain at strain gauge 6, where the strain gauge shows a slightly larger strain. This anomaly might be due to this strain gauge being located where the plies of the root and the blade overlapped. The overlap joint might be causing a slightly lower stiffness in this area. Overall, the strains seem to match well with the numeric predictions.

## 5. Discussion

In this work, a hollow FRP propeller blade, designed to have a particular bend twist deformation during operation, was manufactured. The production was undertaken with a relatively simple method.

An experiment with a point load test was designed and performed to confirm that the manufactured prototype showed the designed bend-twist deformation, i.e., that the prototype had obtained the intended physical/mechanical properties. The manufacturing method and experimental setup were based on simple principles. However, the real-life details and operations in these procedures proved to be quite intricate. Therefore, several details, like staging the load case, numerical modelling of the load case, proposing a comparable boundary condition, designing the propeller blade root, and combining these into one structure with layup tailoring and composite engineering practice were combined with an idealised numeric model from earlier studies to create the realistic prototype.

The manufacturing process included details like mould making, using a silicone rubber bladder to apply pressure from inside the closed split mould, and the ply lapping joint connections between the material layup on the blade’s two sides and between the blade and the root. A 3D scan was done to verify the prototype’s geometric accuracy, which was between –0.5 mm and 1.44 mm, but mainly within a –0.2 to 0.3 mm tolerance. The deviations gave 2.3 mm extra thickness at one location close to the blade trailing edge at propeller radius 0.7, but this was considered negligible for the experiment into the prototypes’ elastic response as it is estimated to only have a minor effect on the structural response in the 600 mm tall propeller blade prototype. The deviation showing an extra thickness of 2.33 mm at the tail was probably caused by too much material in too little volume, which can be solved. Prototypes with higher accuracy could be produced by re-tailoring the laminate, using stiffer moulds, or other state-of-the-art manufacturing methods. Some other minor imperfections were also present in the prototype, but these did not notably affect the prototype structural elastic response.

While the minor imperfections on the prototype might not affect propeller elastic response, they would affect the blade’s marine use. Modern metal propellers can require a surface roughness of less than 10 μm; thus, composite imperfections like pinholes and unforeseen geometry deviation indicate that better manufacturing methods are required when moving towards repeatable production of composite adaptive propeller blades with consistent properties. In addition, the performed 3D scan might work well for confirmation of the outer geometries, but to observe how the fibres were arranged well inside the blade, other methods, like X-rays, are required.

After geometric verification of the manufactured propeller blade, the physical properties were tested. The static load experiment is a test that makes sense in a manufacturing setting as it is straightforward and simple. In this paper, the static test was undertaken by hanging weights from load points to keep the loading direction constant. A downside to using weights was that many weights were needed to match the design loads for the propeller blade. The connection between the hanging weights was simple to stage with a drill and a climbing rope but more challenging to model numerically, so a point load was chosen to be an acceptable simplification.

The experiment was executed, and the deformation of the blade was successfully measured with DIC. The observed deformation characteristics matched the numerical predictions well. The deflection was slightly overestimated at the larger radii, 0.7 and 0.9, but modelled well for the other radii. Whether the deviation observed and predicted was due to the numeric modelling of the load case or numeric modelling of the blade’s mechanical properties is difficult to know, but it is expected that the simplified modelling of the load case affected the results. Nonetheless, the twist was found close to the numerical prediction for the observed deformation.

The mechanical response in the prototype during the experiments was successfully measured with strain gauges. The FEA overestimated the strain slightly compared to the strain gauge data, apart from at a point in the connection between the blade and the root. The higher strain observed here indicates a locally lower stiffness, plausibly due to the ply lapping joint.

Other measurements that would be interesting to explore in the manufacturing process of commercial composite propeller blades are modal analysis to determine the natural frequency, static strength analysis and a spin test of manufactured blade samples on a hub. Methods for testing these properties exist for modern metal propellers. The new feature in composite propellers is the designed elastic response which causes the desired deformation.

Regarding the specifics of the point load experiment, which was meant to mimic the maximum load in a periodic load case, it seems the chosen loading points were not correct. Even though the loading direction was copied, the deformation differed. A plausible hydrodynamic reason could be that the centre of pressure on the blade shifts when the propeller blade stalls under the maximum periodic load. While this is important if it is desired to observe the negative twist in a hydrodynamic setting, the choice of the point load is not critical to assess whether the numerical model deforms like the prototype. The studies [4,22] on comparable tests both showed indications towards similar inaccuracies concerning the magnitude of the blade deflection. Like in [22], the twist of the blade was predicted quite accurately for the point load. When it comes to possible sources of error, several exist in this work. Apart from plausible deviations due to simplified modelling of the experimental load case, like the fact that the load points were located with hand tools and that a rope through the blade was modelled with a point load, the blade model was based on shell elements. Shell elements could influence predicted deformation, especially if the elements are used in laminates that do not adjure to thin shell behaviour. At the same time, the shell elements do not have a volume which means that a shell element’s material volume can overlap with another element’s material volume allowing several materials to exist in the same space. In the numeric blade model, such an overlap occurs along the thin trailing edge, illustrated in Figure 5c. The elements from the two propeller surfaces project elements into the same space, super positioning the elements’ material properties, possibly adding some artificial stiffness.

While all these sources of errors affect the predictions, they are systematic errors rendered as scaling factor which can be considered and reduced through iterating on the FEA and experiment. Nonetheless, as the experiment and FEA correlate to the degree they did, it seems that the modelling choice of using shell elements rather than solid affect the results much less than the expected 14% difference seen in deformation in an FRP propeller blade design when going from 2-way to 1-way FSI, another common modelling choice in propeller blade design [4].

## 6. Conclusions

A 600 mm tall ship propeller blade prototype in CFRP was manufactured. It is based on a propeller blade design that exhibits hydrodynamically desirable bend-twist deformation characteristics. The prototype included a root structure which made it possible to mount the blade to a rigid test rig to perform a point load experiment.

The manufacturing method used to make the prototype was a split mould in combination with a silicone rubber bladder to apply atmospheric pressure to the cast during the high-temperature curing. The CFRP layups applied to each side of the split mould was connected at the mould seam by joint lapping of the plies. The manufactured prototype showed good geometric accuracy with a maximum deviation of 2.3 mm in an area in the tail.

A representative point load experiment mimicking the maximum load in a periodic load case was performed. The experiment was staged physically with an equivalent FEA to verify that the prototype’s FEA predicted properties were obtained. Comparison of experiments and FEA data found that the numeric model shows close to the expected bend and twist deformation, indicating that the modelling captures the reality. At the same time, the FEA slightly overestimated bending deflection and strain while getting the blade twist right, showing that the desired twisting deformation can be built into FRP propellers as a passive mechanical response.

While the production method was sufficient to produce a prototype for an adaptive composite propeller with similar to predicted elastic response, further work is needed to meet the requirements of commercial-grade tolerances to geometric accuracy and surface finish.

The work shows how a static point load test can be undertaken and how to use DIC methods to verify that the sought-after deformation properties exist in a manufactured composite propeller blade. Ultimately, this paper shows that bend-twist designs are both producible and that the sought twist deformation characteristic was similar to the numerical prediction based on a shell model. The desired bend-twist coupling was achieved, and the results show that the numeric model reflects reality.

## Figures and Tables

**Table 1:** Layup of the design in Figure 2. The colours indicate the different CFRP plies. The **red** colour is for backing plies, **black** is for UD plies, and **green** is for surface plies. The 0° orientation is in the green Y direction in Figure 2.

Regions in Figure 2	Number of Plies	Angle Orientation of Plies in the CFRP Layup [°] ^1^
1	15	[**0**, **45**, **0**, **30**, **30**, **0**, **30**, **30**, **0**, **30**, **30**, **0**, **30**, **0**, **45**]
2	18	[**0**, **45**, **0**, **30**, **30**, **0**, **0**, **45**, **30**, **0**, **30**, **0**, **30**, **30**, **0**, **30**, **0**, **45**]
3	13	[**0**, **45**, ** 0**, **0**, **−30**, **−30**, **0**, **−30**, **−30**, **0**, **90**, **0**, **45**]
4	16	[**0**, **45**, ** 0**, **0**, **−30**, **−30**, ** 0**, **0**, ** 45**, **−30**, ** 0**, **−30**, **0**, **90**, **0**, **45**]
5	12	[**0**, **45**, **0**, **−30**, **−30**, **0**, **−30**, **−30**, **0**, **90**, **0**, **45**]

^1^ Outer surface on the right.

**Table 2:** Material properties of the used materials.

Material	E1 [GPa]	E2 [GPa]	E3 [GPa]	ν12	ν13	ν23	G12 [GPa]	G13 [GPa]	G23 [GPa]
Surface ply [0/90]	53	53	7.5	0.05	0.3	0.3	3.5	3.3	3.3
Backing ply [0/90]	58	58	7.5	0.05	0.3	0.3	3.5	3.3	3.3
UD ply [0/90]	117	7.5	7.5	0.34	0.34	0.5	3.5	3.5	3.3

**Table 3:** Calculation of resultant angle and force from the pressure distribution.

Load case	Force Component in X Direction [N]	Force Component in Z Direction [N]	Resultant Force [N]	Resultant Angle (°)
Maximum periodic load	29,460	11,812	21,739	21.8
Minimum periodic load	15,210	10,390	18,419	34.3

**Table 4:** Calculation of blade deflection and twist from the plots in Figure 14.

Propeller Radius	DIC Blade Deflection [mm]	DIC Blade Twist [°]	FEA Blade Deflection [mm]	FEA Blade Twist [°]
0.40	0.783	0.074	0.869	0.097
0.55	3.197	0.138	3.157	0.213
0.70	7.010	0.267	7.575	0.325
0.85	10.832	0.381	12.390	0.415

## Data Availability

The data presented in this study are available within the article.
